# Comparing the diagnostic accuracy of the newly introduced and existing malaria tests in Northwest Ethiopia

**DOI:** 10.1371/journal.pone.0322366

**Published:** 2025-05-07

**Authors:** Tilahun Bizuayehu Demass, Belay Bezabih Beyene, Getasew Mulat Bantie, Melaku Tadege, Agumas Alemu Alehegn, Abraham Amsalu Berneh, Mihretu Jegnie, Mulat Addis Beshaw, Amare Alemu Melese, Wondwossen Amogne Degu

**Affiliations:** 1 Department of Internal Medicine, Infectious Diseases Unit, Addis Ababa University, College of Health Science, Tikur Anbesa Specialized Hospital, Addis Ababa, Ethiopia; 2 Amhara Public Health Institute, Bahir Dar, Ethiopia; 3 Regional Data Management Center for Health, Amhara Public Health Institute, Bahir Dar, Ethiopia; 4 School of Biomedical Sciences, Debre Tabor University, Debre Tabor Town, Ethiopia; 5 Department of Internal Medicine, College of Medicine and Health Science, University of Gondar, Gondar, Ethiopia; 6 Ethiopian Public Health Institute, Addis Ababa, Ethiopia; University of Health and Allied Sciences, GHANA

## Abstract

**Background:**

Malaria is an important public health issue in Ethiopia. Accurate malaria diagnosis plays a vital role in effective treatment. However, there is limited data on the diagnostic accuracy of the newly introduced rapid diagnostic test (LDH-based RDT) and existing diagnostic methods.

**Objective:**

This study aimed to compare the diagnostic accuracy of the LDH-based RDT and existing diagnostic methods among uncomplicated malaria patients, 2024.

**Methods:**

The health-facility-based cross-sectional study was conducted in Dembia and North Achefer districts of the Amhara region. A systematic sampling technique was employed to recruit 460 febrile patients. Each patient’s blood sample was investigated for *Plasmodium* species by RDT, followed by microscopy by different laboratory professionals. The collected blood film slide was sent to the reference laboratory center, Ethiopian Public Health Institute, for confirmatory analysis. The diagnostic accuracy was assessed, and a kappa value > 0.6 was considered as having good agreement between diagnostic techniques.

**Results:**

The sensitivity and specificity of pLDH-based RDT were 99.6 and 100% compared to reference laboratory results. There was a very good consensus between pLDH-based RDT and blood film (Kappa: 0.97, p-value = 0.0001). There was also a good consensus between blood film at the local laboratory and pLDH-based RDT (*Kappa: 0.748, p-value = 0.0001*). However, there was the worst disagreement between clinical diagnosis, pLDH-based RDT, and blood film (*Kappa range: -0.987 to -0.739*).

**Conclusion:**

This study revealed that pLDH-based RDT and microscopy had higher accuracy as per the WHO recommendation standard. This study implied that clinical diagnosis of malaria must be supported either by RDT or microscopy.

## Introduction

Malaria is an infectious protozoal disease caused by *Plasmodium species* [1–3]. It remains an important public health problem worldwide. But the burden is high in developing countries [1,4,5]. In sub-Saharan Africa, most severe cases and deaths occur in children under five years and in pregnant women [5–8].

In the past decade, several African countries, including Libya, Tunisia, Lesotho, and the Seychelles, successfully eradicated malaria. Moreover, Morocco, Algeria, Mauritius, Cabo Verde, and Egypt were certified as malaria-free for their effective diagnostic modalities and intervention strategies [9].

In contrast, the morbidity and mortality of patients from malaria are ever increasing in Ethiopia. In 2022, Ethiopia had contributed a major share to the global increment of malaria cases, and the Amhara region alone reported 960,000 cases, twice as many as the previous year, and this increment of malaria cases is thought to be driven by widespread conflicts, internal displacements, and disruptions in malaria control and prevention programs [10].

The *Plasmodium falciparum* Kelch13 (pfk13) and pfhrp2 mutated plasmodium falciparum (PF) strain is widely spreading in malarious areas [11–13]. The mutation results in both diagnostic resistance that needs urgent evaluation and recommendations for the existing intervention [12–18].

Malaria diagnostic tools are among the components of universal access to malaria prevention strategies to eliminate malaria by 2030 [5,19,20]. Even microscopic and rapid diagnostic tests have less sensitivity and high discordant results compared to standard reference tests [21–30]. In comparative studies of clinical, microscopic, and rapid diagnostic tests for malaria in different African countries, there was a significant discordance among three malaria diagnostic tools [26]. In a meta-analysis of 29 earlier Ethiopian studies on the performance of malaria microscopy and RDT compared to PCR, pLDH-based RDT was found to be more sensitive than microscopy [31,32]. Evidence also showed that the parasite detection performance of *PfHRP2/pLDH CareStart RDTs* was below the WHO standard (>95% at ≥ 100 parasites/μl of blood) [33–35]. It could be linked with the deletion of the *HPR2 gene,* which is an alarming signal for the countries and needs an urgent clinical evaluation and action [36–38]. Thus, in Ethiopia, the recently pfhrp2-based malaria RDT was replaced by species-specific lactate dehydrogenase (*pLDH)-based RDTs*.

Making a malaria clinical diagnosis and confirming the diagnosis with either a rapid diagnostic test or microscopy is recommended in the Ethiopian national malaria treatment guidelines in line with WHO recommendations [3,39,40]. However, in Ethiopia, the majority of malaria diagnoses are still made with clinical diagnosis alone and then treated empirically [41]. Moreover, to the best of our knowledge, there is no single study in Ethiopia that assessed the concordance of clinical microscopy and *pLDH-based RDT* malaria diagnostics.

Therefore, urgent assessment of malaria diagnostic methods of the country will help to adapt and apply plausible *diagnostic algorithms*, thereby avert the drastic consequences of malaria morbidity and mortality, and move forward towards achieving a national malaria elimination program by the year 2030. Thus, this study aimed to assess the performance of clinical, microscopic, and serological malaria diagnostics among uncomplicated malaria patients in Northwest Ethiopia.

## Methods and materials

### Study design, setting, and period

A cross-sectional study was conducted in Dembia and North Achefer districts. from April 1, 2024, to June 30, 2024. These districts are situated in the north Gondar and north Gojjam zones of the Amhara region. Koladiba Health Center is 729 km away from Addis Ababa. While Forhe-Sankira health center is 591 km from Addis Ababa. The peak season for malaria transmission in this region occurs between October and December. Both Plasmodium vivax and falciparum exist in the area [42,43].

### Population

In this study, febrile patients who presented to either Koladiba or Forhe-Sankira health centers for medical attention were the source population, while those patients who visited either of the two health centers and clinically diagnosed malaria patients during the data collection period for febrile case management were the study population.

### Eligibility criteria

In this study, presumed uncomplicated malaria patients of 18 years or older were included, while pregnant or lactating mothers within 6 months post-delivery were excluded.

### Sample size, sampling technique, and procedure

A single population proportion formula was considered, assuming a 50% proportion, a 95% confidence interval, 5% margin of error, and 20% non-response rate. The final sample size was 460. To get the estimated sample size (460), the lists of febrile patients in each health center were proportionally allocated using a systematic random sampling technique. Then, each selected febrile patient was included in the study. At each site, we used a cross-sectional study design to enroll 460 outpatients who had been referred to the laboratory for malaria blood smears in accordance with the usual standard of care at the health centers. Study personnel were not involved in the decision to refer patients to the laboratory. At the time of enrollment, the patient’s sociodemographic data were recorded. A random sample of the febrile patients in both health centers was also submitted to a malaria RDT. The febrile patients submitted to the RDT were screened in a microscope. The reference laboratory test was malaria microscopy executed by highly experienced professionals at the Ethiopian Public Health Institute.

### Data collection tools and procedures

Demographic and clinical details of patients’ data were gathered at the time of registration by enumerators using a pretested questionnaire and data collection checklist. Moreover, blood film microscopy and the serological analysis using lac*tate dehydrogenase-based RDT (pLDH-based RDT)* were done as per the following procedure.

#### Microscopy.

From clinically suspected malaria patients, blood was collected in an EDTA anticoagulated tube for thick and thin blood smears and *pLDH-based RDT* tests. Thin and thick blood films were prepared by experienced medical laboratory technicians using standard operating procedures (SOPs). Thick and thin blood smears were prepared on the same slide for each blood sample. Two drops of blood were placed on a clean, labeled glass slide about 1 cm apart. For the thick blood film, the larger blood spot was stirred in a circular motion with the corner edge of another slide. The thin blood film was prepared by placing the smooth edge of the spreader on the slide the drop of blood at an angle of 45°, and quickly smearing it forward on the slide surface. The blood smears were allowed to air dry, and the thin films were fixed with methanol. The slides were then stained with 10% Giemsa for 10 minutes, after which the stain was washed off and air-dried. Slides were examined using a light microscope with 100x oil immersion, and 100 fields were scanned before a particular smear is declared negative. Parasitemia was calculated for positive blood smears by counting the number of parasites observed per 200 leukocytes and assuming a total of 8,000 leukocytes/µl. Species identification and parasite grading were on thin and thick blood films, respectively. Assuming an average white cell count of 8,000/μl, parasitemia can be estimated from a thick smear by counting the number of parasites until 200 white cells have also been counted. This count, when multiplied by 40, gives an indication of the number of parasites per microliter of blood. The parasitemia percentage can then be calculated by dividing the parasite density by 4,000,000 (the average number of erythrocytes per microliter in blood) and multiplying by 100. The quality of prepared slides was regularly checked by researchers using WHO blood film quality standards, posted at each study site along with SOPs. All slides were taken to and re-examined at a reference laboratory by WHO-accredited experts in the field and were taken as results of the gold standard.

#### RDT.

Similarly, for all presumed malaria cases, serological tests using recently introduced *pLDH-based RDT (BIOCREDIT* Malaria Ag *pf/pv(pLDH/pLDH)*, *Rapigen Inc.,* South Korea) were done from EDTA tube-collected blood according to the manufacturer’s instructions. Each *pLDH-based RDT* device was labeled with the corresponding identification code for each study participant. Approximately 5 μL drop was added into a *pLDH-based RDT* cassette sample well, after which three drops of assay buffer were dispensed into the assay buffer well. The result was read at 25–35 minutes. The results were interpreted following the instructions provided by the manufacturer. A negative result was recorded when only a band appeared in the control area, whereas the presence of two bands, one band in the control area and the other band in the test area, was recorded as a positive result for P. falciparum (PF) or P. vivax (PV) according to the band site. If there are three bands on the control, PF, and PV sites, the test is interpreted as a mixed infection or positive for PF and PV.

Microscopy and *pLDH-based RDT* tests were done separately by different technicians/technologists blindly. When malaria *pLDH-based RDT* is positive but microscopy is negative, treating clinicians are informed by data collectors for treatment decisions.

### Operational definitions

#### Clinically diagnosed malaria.

It is the presumptive diagnosis of patients with malaria-related manifestations [44].

**Confirmed malaria case.** 

A patient with a Blood film or/and RDT positive result.

#### Uncomplicated malaria.

It is a confirmed malaria case without any features of complications or severe malaria, such as diminished consciousness, convulsions, respiratory distress, prostration, hyperparasitemia, severe anemia, hypoglycemia, jaundice, renal insufficiency, hemoglobin urea, shock, cessation of eating and drinking, repetitive vomiting, or hyperpyrexia.

**Concordance.** 

It is the probability that the presumed diagnosis, microscopy, and the RDT test results are the same.

#### Parasite density/parasitemia.

It was categorized as low (< 1000 parasites/µl blood), moderate (1000–4999 parasites/µl blood), high (5000–99,999 parasites/µl blood), and hyper-parasitemia (≥ 100,000 parasites/µl blood) [45–47].

#### Local laboratory.

It is the place where the patients were diagnosed for Plasmodium species (Koladiba or Forhe-Sankira health centers).

#### Reference laboratory.

It was the laboratory found in the Ethiopian Public Health Institute, Addis Ababa.

#### Kappa value.

Kappa values < 0 represent agreement worse than expected or disagreement among raters; however, kappa < 0.20, poor; 0.41–0.60, moderate; 0.61–0.80, good; 0.81–1, very good [48].

### Data quality control

Data completeness and consistency assessments were done on a daily basis throughout the enumeration period.

### Data processing and analysis

The collected data were entered, coded, and cleaned using the EpiData software and analyzed using SPSS version 20. Descriptive statistics were computed and presented in tables and charts. The Chi-Square test was applied to assess the association of parasitic density and each diagnostic method. Pearson’s Chi-Square test was used to evaluate differences in proportions of sensitivity, specificity, positive, and negative predictive values. Determination of agreement between different malaria diagnostic methods was done using Cohen’s kappa test. A kappa value > 0.6 was considered to have good agreement between diagnostic techniques.

### Ethical considerations

The IRB approval was secured by the ethical committee of the Addis Ababa University College of Health Science (with protocol number: 14/23). Written consent was secured from each study subject before commencing data collection. The presentation of results is anonymous, and data were kept confidential. Since there were no minority groups, authors didn’t obtain consent from parents or guardians.

## Results

### Socio-demographic characteristics of participants

A total of 460 patients took part in the study, making the response rate 100%. Of these, 260 (56.5%) were females. The median age of patients was 28 (±26 IQR) years, with the minimum and maximum ages being 18 and 87 years, respectively. Almost three-fourths (74.8%) of study patients were residing in rural areas, and about 41% of them were students. Nearly two-thirds of the cases visited Koladiba health center (Table 1).

**Table 1:** Socio-demographic characteristics of patients with uncomplicated malaria in Northwest Ethiopia, 2024 (n = 460).

Variable	Category	Frequency (%)
Sex	Male	200 (43.5)
	Female	260 (56.5)
Age	15-24 years	243 (52.8)
	25-34 years	106 (23.1)
	35-44 years	76 (16.5)
	≥ 45 years	35 (7.6)
Place of residence	Rural	344 (74.8)
	Urban	116 (25.2)
Occupation	House wife	134 (29.1)
	Farmer	97 (21.1)
	Merchant	29 (6.3)
	Government employee	4 (0.9)
	Student	190 (41.3)
	Daily laborer	6 (1.3)
Study site	Koladiba Health Center	300 (65.2)
	Forhe-Sankira Health Center	160 (34.80

### Clinical diagnosis of malaria

In this study, the most presenting complaints were fever (100%) and headache (100%). In terms of vital signs, nearly all patients (99%) exhibited normal blood pressure and respiratory rates, and none of the patients required artificial ventilation. However, almost all patients were tachycardic and febrile (Table 2).

**Table 2:** Clinical diagnosis of patients with uncomplicated malaria in Northwest Ethiopia, 2024 (n = 460).

Variable	Category	Frequency (%)
Blood pressure (mmHg)	90-139/80-89	456 (99.1)
	>140/90	4 (0.9)
Heart rate	Normal	7 (1.5)
	Tachycardic (>100 bpm)	453 (98.5)
Respiratory rate	Normal	453 (98.5)
	Tachypneic	7 (1.5)
O_2_ saturation	Maintained at room air	460 (100)
Axillary temperature	Febrile (>37.5°C)	460 (100)
Signs of anemia	No	448 (97.4)
	Yes	12 (2.6)

### Laboratory diagnosis of malaria

The current study showed that malaria diagnosis was performed using blood films and rapid diagnostic tests by both a laboratory technician (86.1%) and a laboratory technologist (13.9%) at the research site laboratory. Most participants (60%) had between three and five years of work experience, and nearly half of the laboratory staff had received training in malaria diagnosis. Microscopic analysis showed that 50.7% of clinically suspected malaria cases were positive, while 48.9% tested positive using RDT. Although both methods confirmed positive malaria cases, there was a five percent difference (23 out of 460) in identifying Plasmodium species. More than one-third of the patients had a parasite density ranging from 5,000–100,000 parasites per µl of blood (Table 3).

**Table 3:** Blood film and rapid diagnostic test results of malaria among patients with uncomplicated malaria in Northwest Ethiopia, 2024 (n = 460).

Variables	Category	Frequency (%)
PLDH-based RDT result (n = 460) and *Plasmodium* species identified (n = 226)	Negative	234 (50.9)
	Positive	226 (49.1)
	*Plasmodium falciparum (PF)*	150 (66.4)
	*Plasmodium vivax (PV)*	68 (30.1)
	*Mixed (PF + PV)*	8 (3.5)
Blood film result at local laboratory (n = 460) and *Plasmodium* species reported (n = 234)	Negative	226 (49.1)
	Positive	234 (50.9)
	*Plasmodium Falciparum*	146 (62.4)
	*Plasmodium Vivax*	78 (33.3)
	*Mixed (PF + PV)*	10 (4.3)
Blood film result at reference laboratory and *Plasmodium* species reported	Negative	233 (50.7)
	Positive	227 (49.3)
	*Plasmodium falciparum*	150 (66.1)
	*Plasmodium vivax*	70 (30.8)
	*Mixed (PF + PV)*	7 (3.1)
Parasitic density/µl (at reference laboratory)	1000 parasites/µl blood	8 (1.7)
	1000-4999 parasites/µl blood	28 (6.1)
	5000–99,999 parasites/µl blood	174 (37.8)
	≥ 100,000 parasites/µl blood	15 (3.3)

### Malaria diagnostic techniques

The current study reported Plasmodium falciparum, Plasmodium vivax, and mixed species were identified using microscopy (in both local and reference laboratories) and pLDH-based rapid diagnostic tests (RDTs). The average parasite density observed through microscopy was 16,229.41 ± 10,533.62 parasites/ µ L. Considering the blood film analysis at the reference laboratory as the diagnostic gold standard, the pLDH-based RDT demonstrated a sensitivity of 99.6% and a specificity of 100%. In contrast, the blood film analysis performed at the local laboratory also exhibited a sensitivity of 99.6% but a specificity of 96.6%. Both the pLDH-based RDT and the blood film from the local laboratory depicted similar negative predictive values (NPV) of 99.6%, while the positive predictive value for the local laboratory’s blood film was lower compared to that of the reference laboratory’s blood film (Table 4).

**Table 4:** Diagnostic accuracy of different malaria diagnostic methods in the northwest, Ethiopia (n = 460).

Parameter	Sensitivity (%)	Specificity (%)	PPV (%)	NPV (%)
BF at local laboratory	99.6	96.6	96.7	99.6
PLDH-based RDT	99.6	100	100	99.6
Clinical diagnosis	49.13	49.13	49.13	49.13

The current study found a significant relationship between parasitic density and the blood film results at the local laboratory (X² = 18.24, p < 0.0001). Additionally, while higher levels of parasitic density were linked to an increased chance of accurately diagnosing malaria using the pLDH-based RDT, this relationship was not statistically significant (X² = 0.93, p = 0.817) (Table 5).

**Table 5:** Relationship between malaria diagnostic methods and parasite density (n = 460).

Parameter	Status	Parasite density/µl blood				X^2^, P-value
		< 1000 parasites	1000-4999 parasites	5000–99,999 parasites	≥ 100,000 parasites	
BF at local laboratory	Negative	4 (50%)	22 (78.6%)	159 (91.4%)	15 (100%)	18.24, 0.0001
	Positive	4 (50%)	6 (21.4%)	15 (8.6%)	0 (0%)	
RDT Result	Negative	0 (0%)	1 (3.6%)	3 (1.7%)	0 (0%)	0.93, 0.817
	Positive	8 (100%)	27 (96.4%)	171(98.3%)	15 (100%)	

### Diagnostic performance of test methods

The current study showed that the level of agreement estimated between the different diagnostic methods for malaria was varied. The weighted kappa showed a very good consensus (*Kappa: 0.969, p-value = 0.0001*) between BF at the reference laboratory and BF at the local laboratory, as well as between BF at the reference laboratory and *pLDH-based RDT* (*Kappa: 0.97, p-value = 0.0001*). Moreover, there was also a good consensus between BF at the local laboratory and pLDH-based RDT (*Kappa: 0.748, p-value = 0.0001*). However, there was a significantly worse disagreement between clinical diagnosis and pLDH-based RDT as well as clinical diagnosis and microscopy (local and reference laboratory) (*Kappa range: -0.987 to -0.739*) (Table 6).

**Table 6:** Level of agreement between different diagnostic methods for malaria (n = 460).

Diagnostic methods	P-value	kappa statistic
BF at the reference laboratory and pLDH-based RDT	0.0001	0.97
BF at reference laboratory and BF at local laboratory	0.0001	0.969
BF at the local laboratory and pLDH-based RDT	0.0001	0.748
Clinical diagnosis and pLDH-based RDT	0.0001	-0.752
Clinical diagnosis and BF at the reference laboratory	0.0001	-0.739
Clinical diagnosis and BF at the local laboratory	0.0001	-0.987

## Discussion

The current study showed the diagnostic concordance of the currently available malaria diagnostic methods: clinical diagnosis by a physician, microscopy, and pLDH-based RDT. This study showed that four major malaria non-specific symptoms were reported by the patients as documented in the patients’ chart. In this study, the most presenting complaints were fever (100%) and headache (100%). This finding was in line with most studies of Ghana, North India [25,49,50]. This finding was in line with different studies that high-grade fever was associated with high parasitic index [49,51]. Moreover, regarding headaches in malaria, scholars showed that pro-inflammatory cytokines such as TNF-α and IL-6 have an important role in the pathogenesis of headaches in malaria [50]. Thus, clinical diagnosis showed generally low sensitivity (49.13%) and specificity (49.13%) in comparison with the gold standard (BF at referral laboratory). The low specificity implies that though the signs and symptoms might be due to other febrile illnesses, the clinicians might diagnose malaria without laboratory confirmation and treat the patients with antimalarial drugs. This may give a high number of false positives for malaria, even when it is not actually present. This suggested that the practice of clinical diagnosis and initiating antimalarial drugs without laboratory diagnosis could lead to either resistance to *Plasmodium species* or mismanagement of other febrile illnesses and revealed the necessity of diagnosis by microscopy by well-trained laboratory professionals [25,52]. The current study’s clinical diagnosis finding was different from the study findings of the Ho Municipal Hospital in the Volta Region of Ghana (25.82%) and in four districts in the Eastern Region, Ghana (80%), respectively [25,53]. But it was lower than the Nigerian study finding, for which the specificity was 95.93% [54]. The possible justifications for this finding could be the sample size, the study setting, the period, and the design.

Using microscopy at the reference laboratory as the gold standard, pLDH-based RDTs have demonstrated varying degrees of diagnostic accuracy for malaria infections. In this study, PLDH-based RDT exhibited a sensitivity of 99.6% and a specificity of 100%. This contrasts with findings from Ghana, where sensitivity and specificity were reported at 91.20% and 25.0%, respectively [25,55,56]. The sensitivity observed in this study aligns with the WHO’s recommended standard of 95% sensitivity for P. falciparum densities of 100/μL and a specificity of 95% for an acceptable malaria RDT [57]. Research indicates that RDT performance can be affected by factors such as low parasite density, storage temperature, and humidity, which may pose challenges in accurately identifying parasites at the species level or quantifying infection density [58]. Moreover, the diagnostic performance of HRP-based RDT is highly compromised in Ethiopia due to the emergence of diversity PfHRP2 and PfHRP3 amino acid repeat including novel repeats. These results indicate that there is a need to closely monitor the performance of PfHRP2 RDT associated with the genetic variation of the pfhrp2 and pfhrp3 gene in P. falciparum isolates at the country-wide level and paves the wat for the introduction of pLDH-based RDT in the Ethiopian health system [59,60].

Compared with the microscopy at the reference laboratory, pLDH-based RDT demonstrated both a high positive predictive value (100%) and a negative predictive value (99.6%). However, the former studies showed lower negative predictive values, implying that a test result by RDT may indicate a false negative result by showing that an infection is absent while present [55,56,61,62]. Moreover, in the current study, 3 (1.7%) patients confirmed RDT negative and had a recorded parasitic density between 5,000 and 99,999 parasites/μL.

The current study found a significant relationship between parasitic density and the blood film results at the local laboratory (X² = 18.24, p < 0.0001). Moreover, while higher levels of parasitic density were linked to an increased chance of accurately diagnosing malaria using the pLDH-based RDT, this relationship was not statistically significant (X² = 0.93, p = 0.817). This finding is supported by study findings of Uganda, Mozambique, and Tanzania [63,64].

The weighted kappa analysis demonstrated a strong consensus (Kappa: 0.969, p-value = 0.0001) between blood film (BF) results from the reference laboratory and those from the local laboratory. Moreover, there was also a strong association between BF results from the reference laboratory and rapid diagnostic tests (RDT) (Kappa: 0.97, p-value = 0.0001). This suggests that either diagnostic method is sufficient for diagnosing malaria infection. These results align with studies done in Cameroon [65] and Ghana [53]. However, the current findings showed a poor level of agreement with another study from Ghana, which reported a kappa value of 0.087 [25].

There was also a significant disagreement between clinical diagnoses and pLDH-based RDT test results. In addition, there was a disagreement between clinical diagnoses and microscopy results from both local and referral laboratories (Kappa range: -0.987 to -0.739). This suggests that clinical diagnoses should be supplemented with either RDT or microscopy in the study area. Initiating therapy relying solely on clinical evaluation has lower sensitivity and could lead to misdiagnosis of malaria and be attributed to drug resistance [25,66].

### Limitations of the study

The authors used the purposive sampling technique to recruit districts and health centers, so it was difficult to make inferences. Moreover, the study solely included patients older than 18 years; this might make it difficult to generate comprehensive evidence for the entire population.

## Conclusion

This study revealed that the pLDH-based RDT and microscopy had higher accuracy as per the WHO standard. Diagnosing malaria by microscopy or RDT has the potential to accurately identify the infection.

## Supporting information

S1 Data SPSS version dataset. (SAV)

## Abbreviations

BF: Blood Film
BF: Blood Film
BPM: beats per minute
EDTA: Ethylene Diamine Tetra Acetic Acid
IQR: Interquartile Range
IRB: Institutional Review Board
MMHG: millimeters mercury
OR: Odds ratio
PCR: Polymerase Chain Reaction
*PF*: *Plasmodium falciparum*
*PV*: *Plasmodium vivax*
RDT: Rapid Diagnostic Test.
